# Coronavirus Disease 2019 (COVID-19) in a Renal Transplant Patient

**DOI:** 10.7759/cureus.8038

**Published:** 2020-05-09

**Authors:** Avantika Chenna, Venu Madhav Konala, Vijay Gayam, Srikanth Naramala, Sreedhar Adapa

**Affiliations:** 1 Nephrology, Phoebe Putney Memorial Hospital, Albany, USA; 2 Nephrology, Medical College of Georgia, Augusta, USA; 3 Hematology and Oncology, Ashland Bellefonte Cancer Center, Ashland, USA; 4 Hematology and Oncology, King's Daughters Medical Center, Ashland, USA; 5 Internal Medicine, Interfaith Medical Center, New York, USA; 6 Rheumatology, Adventist Medical Center, Hanford, USA; 7 Nephrology, Kaweah Delta Medical Center, Visalia, USA

**Keywords:** coronavirus, acute respiratory distress syndrome, covid-19, renal transplant

## Abstract

Coronavirus disease 2019 (COVID-19) has resulted in significant morbidity and mortality worldwide. Transplant patients are particularly at a higher risk of contracting COVID-19 because of their immunosuppressed state, and they have the propensity to develop opportunistic infections. The pre-immunosuppressed state, along with other existing comorbidities, can influence the outcomes of COVID-19 in transplant patients. We describe a case of a renal transplant patient who developed COVID-19. Real-time nucleic acid testing (NAT) should be done in deceased and living donors. The most common management strategy is the modification of immunosuppression along with current experimental strategies for COVID-19.

## Introduction

The pandemic of coronavirus disease 2019 (COVID-19) is a public health emergency caused by the novel coronavirus, which is also termed as severe acute respiratory syndrome coronavirus 2 (SARS-CoV-2). Transplant patients are particularly at a higher risk of contracting COVID-19 because of their immunosuppressed state, and they have the propensity to develop opportunistic infections [1]. The pre-immunosuppressed state, along with other existing comorbidities, can influence the outcomes of COVID-19 in transplant patients. Here, we describe a case of a renal transplant patient who developed COVID-19 and, unfortunately, died from the infection despite all medical management.

## Case presentation

A 54-year-old African American male presented with fever, cough, and weakness for two weeks duration. The patient denied having any chest pain, shortness of breath, nausea, vomiting, or diarrhea. The patient denied any travel history, contact with any person tested positive for COVID-19, or attending any public gatherings. The patient was admitted to the hospital two weeks before the presentation for left lower extremity superficial femoral artery angioplasty and could have likely got exposed to COVID-19.

Past medical history included end-stage renal disease (ESRD) secondary to diabetic nephropathy and underwent deceased donor kidney transplant in 2016, hypertension, diabetes mellitus, peripheral vascular disease status post right above knee amputation (AKA). The patient was taking tacrolimus 5 mg twice a day, mycophenolate mofetil (MMF) 1000 mg twice a day, and prednisone 5 mg daily for maintenance immunosuppression. Other home medications included simvastatin 10 mg daily, lisinopril 20 mg PO daily, insulin Humalog sliding scale before meals, insulin Levemir 30 units daily, clopidogrel 75 mg PO daily, and gabapentin 75 mg PO daily.

On presentation, the patient was febrile with 39.4 Celsius, pulse rate 109 beats per minute, blood pressure 114/72 mm Hg, respiratory rate 19 breaths per minute, and oxygen saturation 95% on room air. Physical examination was significant for a patient in respiratory distress with decreased breath sounds on bibasilar lung fields. The rest of the physical examination was unremarkable. The labs were summarized in Table 1. The patient’s baseline creatinine was 2.0 gm/dl with sub-nephrotic range proteinuria on the labs done three months ago.

**Table 1:** Summary of laboratory testing BUN: blood urea nitrogen; COVID-19: coronavirus disease 2019; NAA: nucleic acid amplification; PCR: polymerase chain reaction

Parameters	Reference range	Day 1	Day 17
Hemoglobin	11-15 (g/dl)	11.6	8.5
Hematocrit	35-46 (%)	37.7	27.6
White blood cell count	4.5-11 (10^3^/uL)	4.3	13.1
Lymphocytes	22-48 (%)	3	Not available
Neutrophils	40-70 (%)	86	Not available
Platelet count	150-450 (10^3^/uL)	146	216
Sodium	136-145 (mmol/L)	138	148
Potassium	3.5-5.1 (mmol/L)	5.8	3.8
Bicarbonate	23-31 (mEq)	20	33
BUN	9.8-20.1 (mg/dl)	62	59
Creatinine	0.57-1.11 (mg/dl)	3.68	2.36
Phosphorus	2.3-4.7 (mg/dl)	3.1	3.7
Magnesium	1.6-2.6 (mg/dl)	1.6	2.4
Creatine kinase	29-168 (U/L)	73	51
Ferritin	30-400 (ng/ml)	2724.0	2645.7
C-reactive protein	0-10 (mg/L)	8.7	36.7
Erythrocyte sedimentation rate	0-20 (mm/hr)	Not available	111
Lactate dehydrogenase	125-220 (U/L)	291	370
Troponin I	0.00-0.03 (ng/ml)	0.034	0.04
D-dimer	0-500 (ng/ml)	Not Available	2.31
B-natriuretic peptide	10-100 (pg/ml)	28	Not available
Interleukin -6	0.0-15.5 pg/mL	Not available	Not available
Urine toxicology		Negative	
Tacrolimus level	ng/ml	4.7	7.2
Influenza	Type A antigen type B antigen	Negative	
COVID-19	NAA/PCR	Positive	

**Figure 1:**
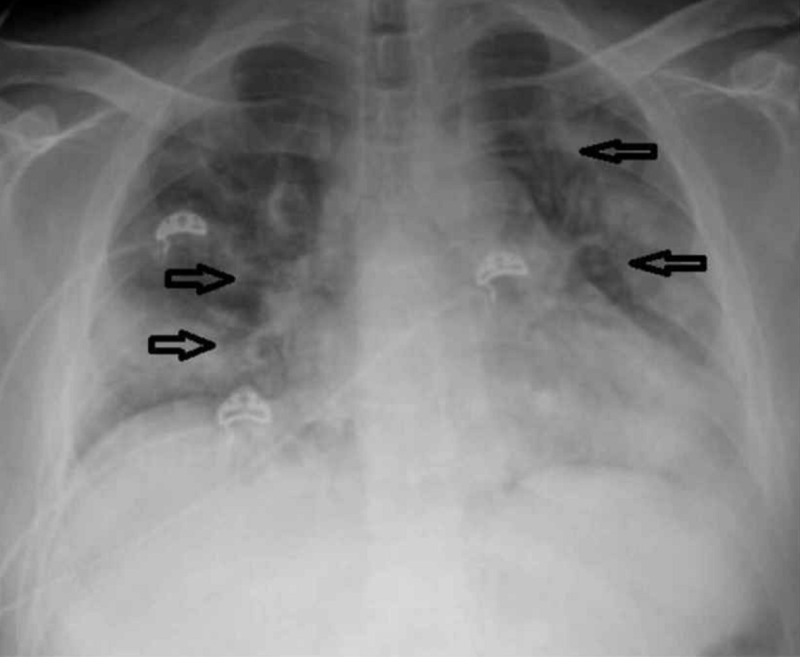
Chest X-ray portable revealed cardiomegaly with bilateral lung infiltrates

**Figure 2:**
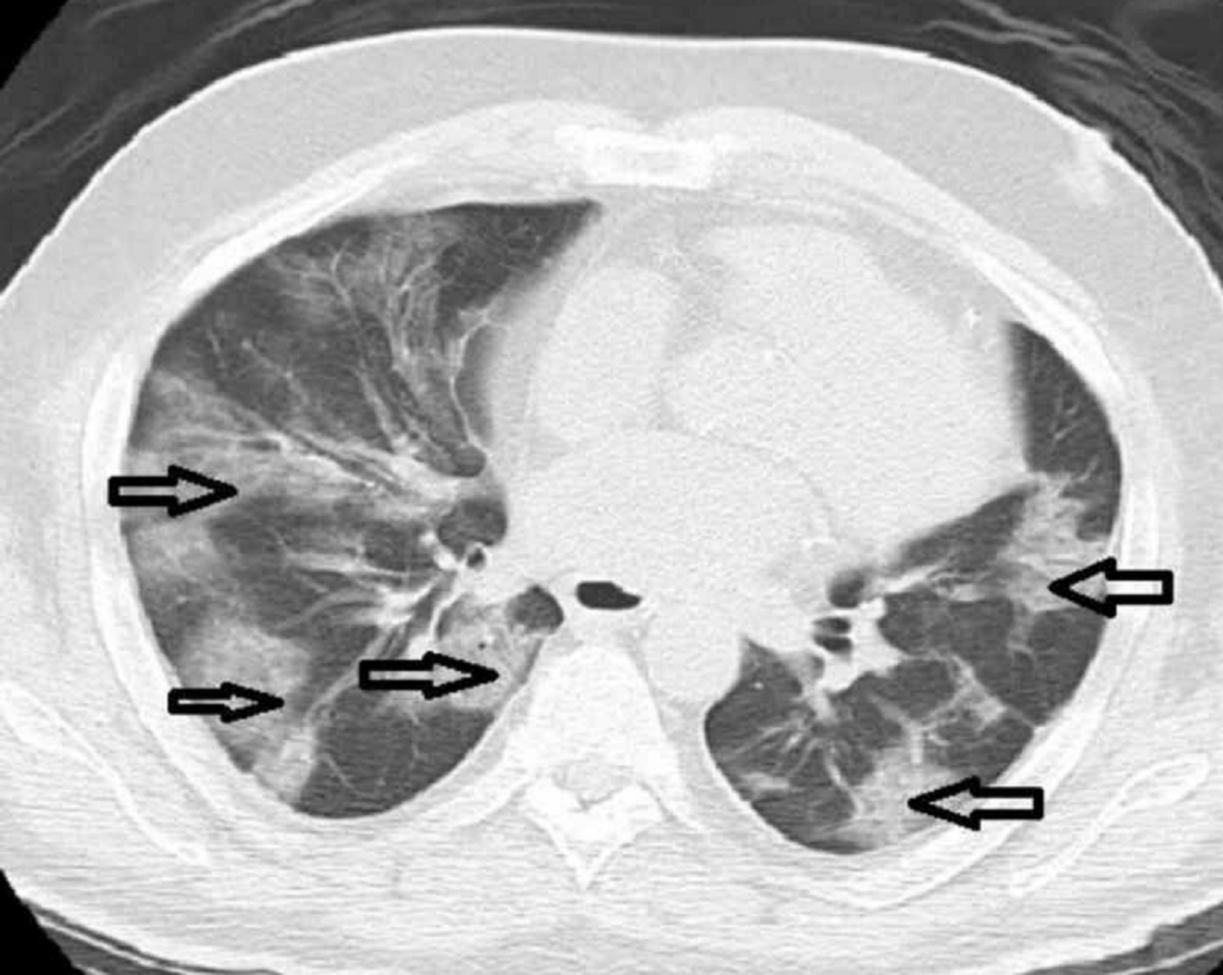
Computed tomography (CT) of the chest without contrast revealed prominent multifocal pneumonia and multiple ground-glass airspace opacities throughout all lung fields

The chest X-ray revealed cardiomegaly with bilateral lung infiltrates (Figure 1). Computed tomography (CT) of the chest without contrast revealed prominent multifocal pneumonia and multiple ground-glass airspace opacities throughout all lung fields (Figure 2).

**Figure 3:**
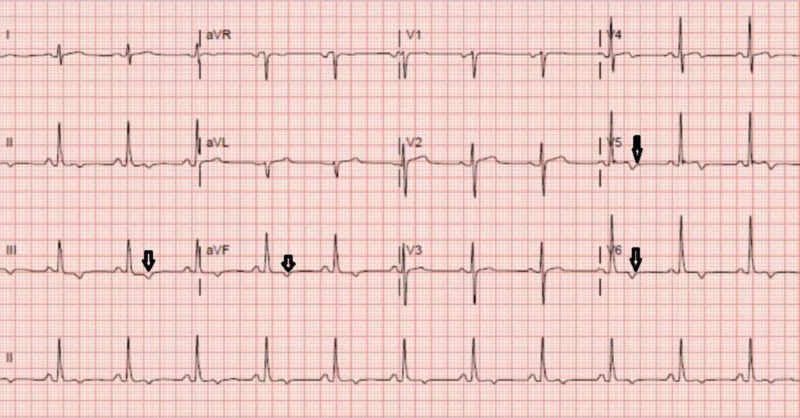
EKG before starting hydroxychloroquine and azithromycin showing sinus rhythm at 75 beats/minute, mild t-wave inversion in inferior and lateral leads, normal QTc interval EKG: electrocardiogram

**Figure 4:**
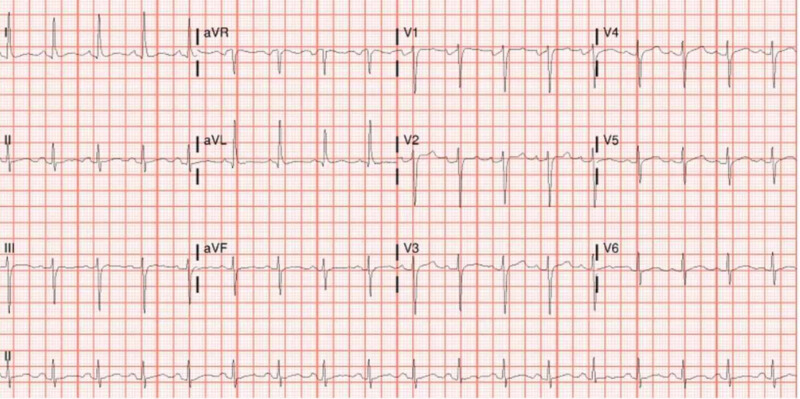
EKG after starting hydroxychloroquine and azithromycin showing sinus rhythm at 100 beats/minute and normal QTc interval EKG: electrocardiogram

**Figure 5:**
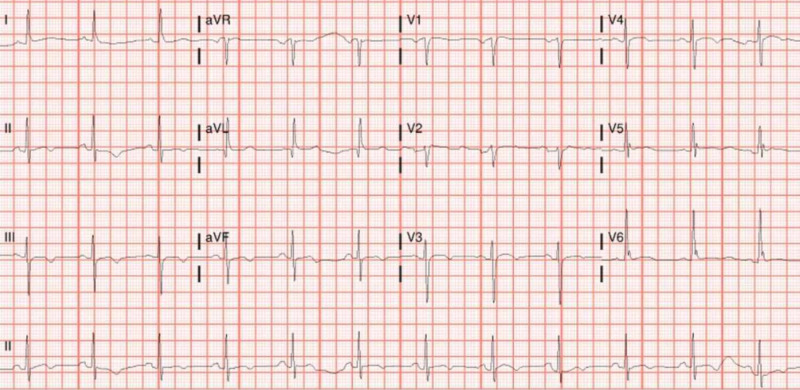
EKG on the final day of hydroxychloroquine and azithromycin showing normal sinus rhythm at 75 beats per minute, non-specific t-wave abnormality and normal QTc interval EKG: electrocardiogram

The patient was started on treatment with hydroxychloroquine, azithromycin, and ceftriaxone for suspected COVID-19 and pneumonia. Electrocardiogram (EKG) was done every 48 hours to monitor the QTc interval. All EKGs showed a normal QTc interval (Figures 3-5). The MMF, prednisone, and lisinopril were held, tacrolimus was continued at a home dose, and the patient was started on methylprednisolone 50 mg every eight hours. Tacrolimus was stopped within 72 hours, as the patient continued febrile and hypotensive. The patient was started on norepinephrine for hypotension and required continued escalation of treatment with three pressors.

The patient also developed intermittent episodes of acute kidney injury, which was managed with intravenous fluids and diuretics as needed but never needed dialysis. The respiratory status deteriorated over the hospital course, progressing to acute respiratory distress syndrome requiring intubation and ventilation with 100% fraction of inspired oxygen (Fio2) and high positive end-expiratory pressure. Unfortunately, the patient developed asystole and died despite resuscitative efforts 17 days into hospital admission.

## Discussion

COVID-19 is a rapidly evolving disease with a high transmission rate and has changed our lives in an unprecedented way. The transplant patients are a special group that needs collaborative effort amid this global health crisis. The viral load and mortality were higher in transplant recipients infected with prior coronavirus outbreaks [2]. The illness from COVID-19 in renal transplant recipients ranged from mild to severe, and few patients presented with atypical symptoms [1,3].

There were no reported instances that COVID-19 was spread through organ donation [2]. The detection of SARS-CoV-2 in blood and organs ascertains that transmission through organ donation is a possibility [4]. The transplant societies have issued guidance worldwide on screening the donors and recipients to decrease the spread [2]. Real-time nucleic acid testing (NAT) should be done in deceased and living donors [2]. Taking universal precautions and appropriate personal protective equipment (PPE) should be used to decrease the risk of transmission during organ procurement [2]. The transplant surgeries should be postponed if there is known exposure. The decision to proceed with transplant surgery should be individualized based on the risk and benefits of proceeding with transplantation and the introduction of immunosuppression. The transplant recipients and the care team members should follow the same precautions as the general public in the event of exposure or development of symptoms.

Management is challenging because there is no proven drug therapy and the experience is based on a few studies. The common practice is to modify immunosuppression by holding mycophenolate mofetil/azathioprine and adjusting the dose of calcineurin inhibitors (CNIs), such as tacrolimus, in our patient [5]. In a study by the University of Washington with 210 patients (150 renal transplant patients), 73% have a reduction in immunosuppression anti-metabolite, held in almost all patients [5]. Drug interactions should be paid close attention to as reported by Bartiromo [6]. Higher-dose steroids were used, which can be associated with delayed viral clearance [7].

## Conclusions

Transplant patients constitute a population more vulnerable to develop COVID-19 because of their immunosuppressed state and higher risk for opportunistic infections. Management includes the modification of immunosuppression with anti-metabolite held in most patients. Prevention is the key, as there is no proven treatment or vaccine available. We advise caution while using high-dose steroids, as it can be associated with delayed viral clearance.
